# Oxidative atmosphere-driven formation of single-phase spinel CuRh_2_O_4_ nanofibers for alkaline water oxidation

**DOI:** 10.3762/bjnano.17.50

**Published:** 2026-05-27

**Authors:** Namhee Kim, Sumin Ko, Sohyeon Choi, Seoyoon Jang, Myung Hwa Kim, Dasol Jin

**Affiliations:** 1 Department of Chemistry and Nanoscience, Ewha Womans University, Seoul 03760, Republic of Korea; 2 Department of Chemistry, Jeonbuk National University, Jeonju 54896, Republic of Korea; 3 Institute of Multiscale Matter and System (IMMS), Ewha Womans University, Seoul 03760, Republic of Korea

**Keywords:** annealing, electrocatalyst, electrospinning, oxygen evolution reaction, spinel oxide

## Abstract

Cu–Rh bimetallic single-phase spinel oxide nanofibers were synthesized via electrospinning followed by post-annealing in precisely controlled oxidative environments. By systematically tuning the O_2_ concentration in the He carrier gas flow during the annealing process, the optimal atmosphere was identified to produce phase-pure CuRh_2_O_4_. The as-prepared CuRh_2_O_4_ nanofibers exhibited excellent electrocatalytic performance toward the oxygen evolution reaction in 1.0 M NaOH (aq), highlighting the importance of atmosphere-controlled thermal treatment for engineering high-activity spinel oxide electrocatalysts.

## Introduction

The oxygen evolution reaction (OER) is a kinetically demanding, multistep process that governs the efficiency of alkaline water electrolysis [1]. Developing robust and highly active OER electrocatalysts is therefore essential for practical hydrogen production and large-scale renewable energy conversion. Among numerous catalyst platforms, spinel oxides (AB_2_O_4_) have attracted significant attention due to their structural robustness and compositional tunability [2]. The spinel framework accommodates diverse metal cations with flexible site occupancy, enabling rational modulation of electronic structure and surface adsorption energetics of key OER intermediates [3], thereby offering a versatile strategy for performance optimization. Incorporation of 4d transition metals such as Rh into the spinel lattice provides an effective approach to further tailor catalytic properties. The presence of Rh^3+^ [4–5], with its more delocalized electronic structure compared to conventional 3d cations, enhances metal–oxygen (M–O) covalency and optimizes the adsorption energies of key intermediates (*OH, *O, and *OOH), leading to improved reaction energetics [6–8].

Despite these advantages, synthesizing phase-pure spinel oxides remains challenging when Cu is incorporated. Cu-based catalysts are particularly sensitive to the synthetic environment because Cu readily changes its oxidation state (Cu^0^/Cu^+^/Cu^2+^) depending on the oxidative atmosphere during annealing [9–10]. As a result, slight variations in oxygen partial pressure can significantly alter phase evolution and often lead to undesired secondary phases (e.g., CuO or Cu_2_O) [11]. Thus, establishing an atmosphere-controlled synthesis route is critical for producing single-phase Cu-containing spinel oxides with reliable and optimized electrocatalytic properties.

Herein, we demonstrate the synthesis of Cu–Rh bimetallic single-phase spinel oxide nanofibers via electrospinning followed by post-annealing under precisely controlled oxidative environments. By deliberately controlling the annealing atmosphere under the continuous O_2_/He flow, optimized conditions were identified to obtain single-phase CuRh_2_O_4_ nanofibers. The resulting spinel oxides exhibit excellent OER electrocatalytic activity in 1 M NaOH (aq), highlighting the importance of oxygen-atmosphere engineering for the rational design of Cu-based spinel oxide catalysts.

## Results and Discussion

**Figure 1:**
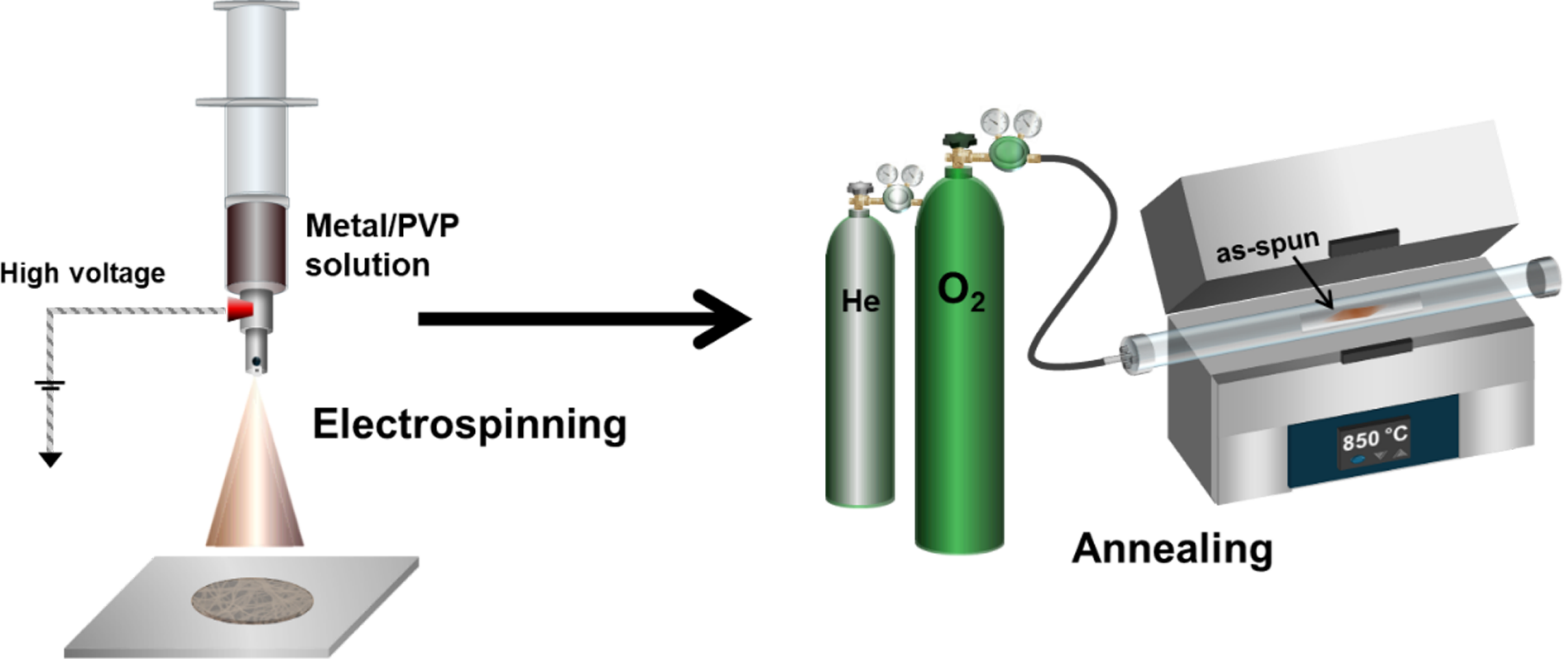
Schematic illustration of the synthesis procedure used in this study.

A series of Cu–Rh bimetallic oxide nanofibers were synthesized via electrospinning and a subsequent annealing process under continuous O_2_/He flow, as illustrated in Figure 1 (see Experimental section for details).

**Figure 2:**
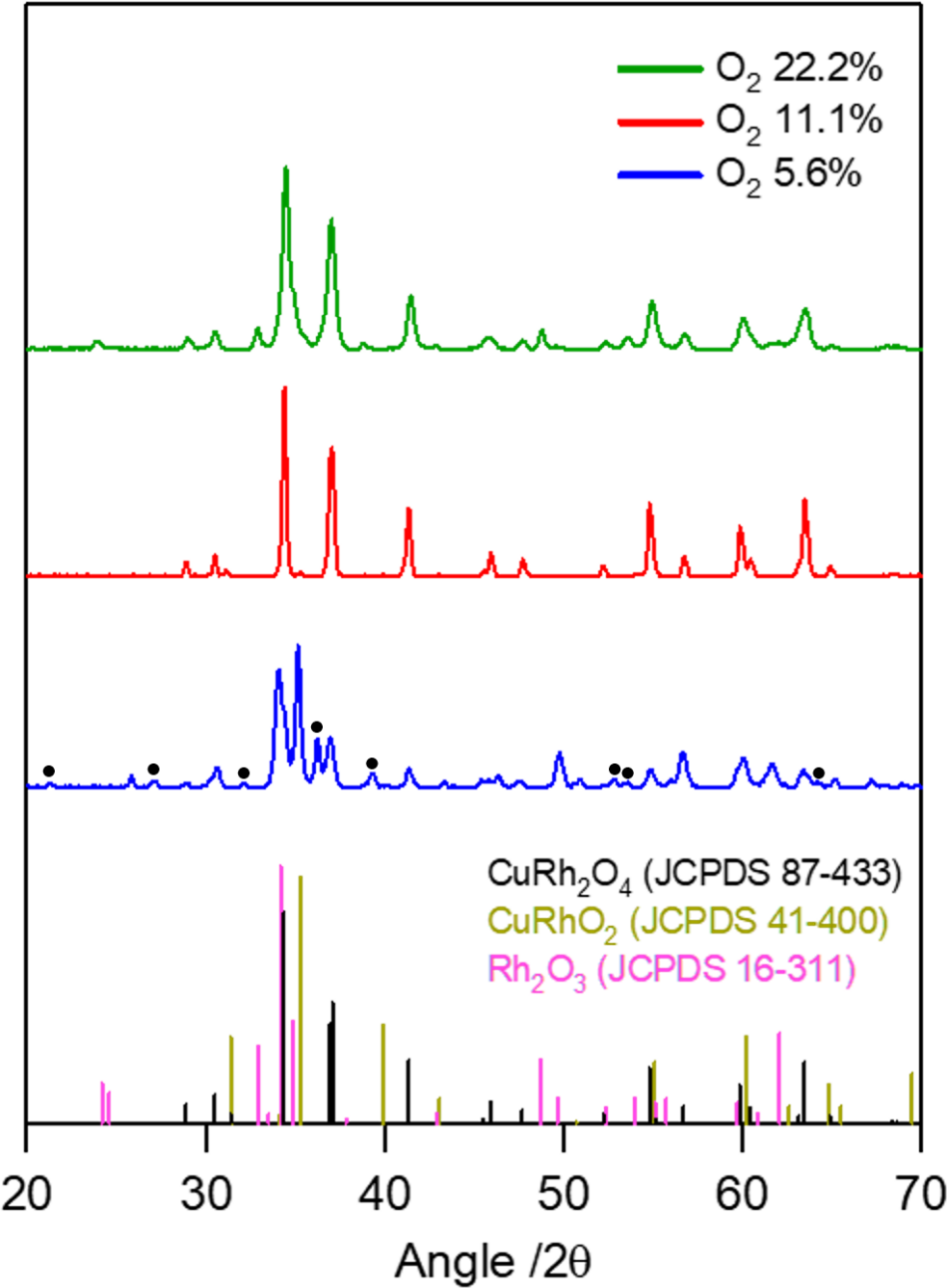
XRD patterns of the as-prepared nanomaterials synthesized under different O_2_ concentrations. Peaks marked with • indicate the presence of secondary copper oxide phases (Cu_2_O).

As shown in Figure 2, X-ray diffraction (XRD) analysis was employed to investigate the phase evolution of Cu–Rh oxide nanofibers annealed under different oxidative environments (i.e., O_2_ concentrations of 5.6%, 11.1%, and 22.2%). The diffraction patterns reveal that the O_2_ concentration during annealing plays a decisive role in determining the crystallographic phase and phase purity. Under insufficiently oxidative conditions (i.e., 5.6% O_2_), additional patterns attributable to CuRhO_2_, Rh_2_O_3_, Rh, and Cu_2_O are observed, indicating incomplete formation of the targeted spinel structure. In contrast, the optimized oxygen concentration (i.e., 11.1% O_2_) yields diffraction peaks that can be fully indexed to spinel CuRh_2_O_4_, confirming the formation of a single-phase crystalline structure. However, under excessively oxidative conditions (i.e., 22.2% O_2_), mixed phases consisting of CuRh_2_O_4_ along with CuRhO_2_ and Rh_2_O_3_ are observed. A similar trend in phase evolution and separation of Cu–Rh bimetallic oxides was observed under an alternative annealing condition of 3 h, while all other synthesis parameters were kept identical to those described above (Supporting Information File 1, Figure S1). These results highlight that phase evolution is governed by the interplay between overall oxygen concentration and transient local redox environments during precursor decomposition, and that precise oxygen-atmosphere engineering during annealing is critical for suppressing undesired phase segregation and achieving phase-pure CuRh_2_O_4_ nanofibers.

**Figure 3:**
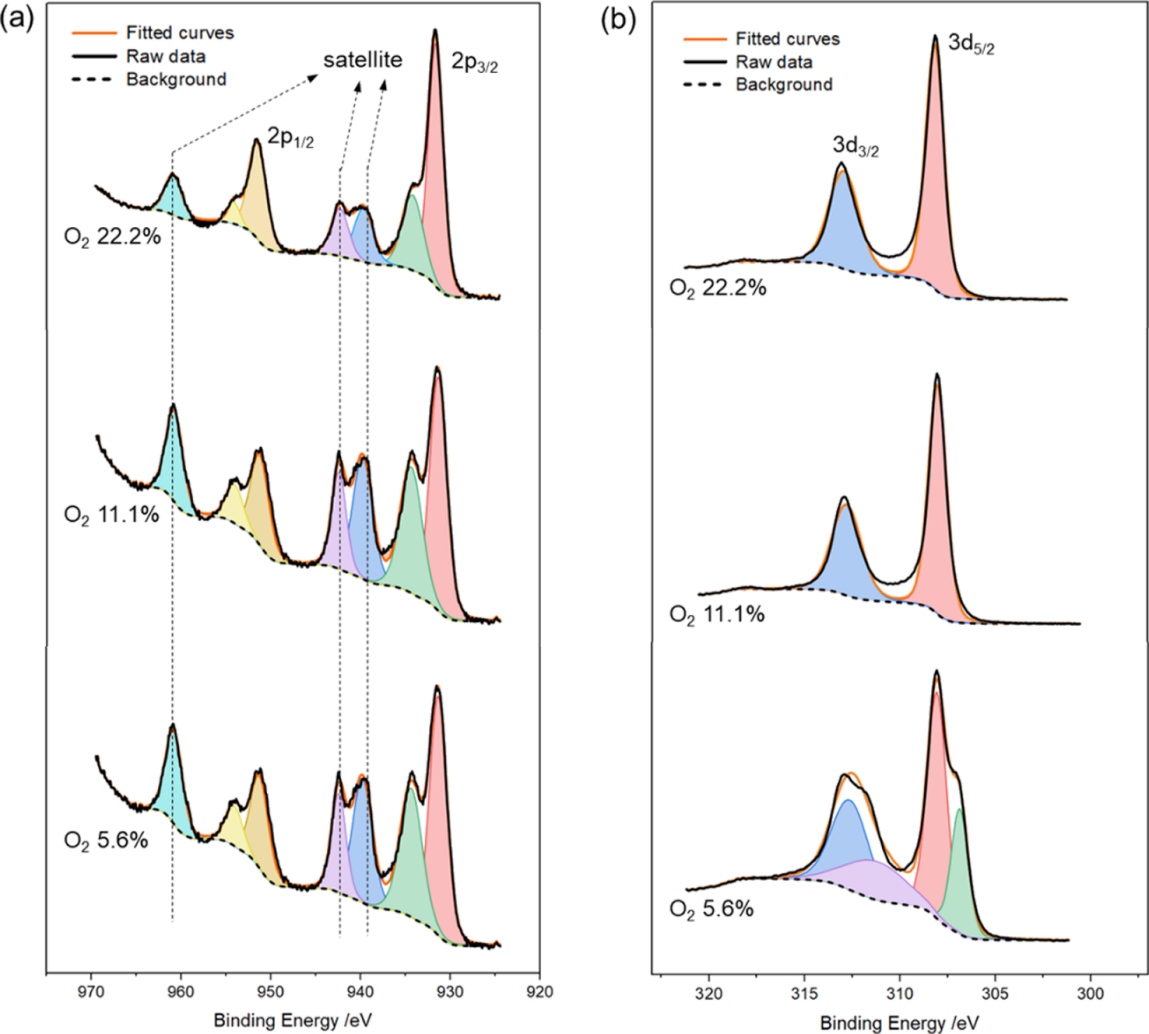
Deconvoluted AR-XPS spectra of Cu–Rh bimetallic oxides in the (a) Cu 2p and (b) Rh 3d regions.

Angle-resolved X-ray photoelectron spectroscopy (AR-XPS) was performed to clarify the surface chemical states of Cu and Rh in Cu–Rh bimetallic oxides synthesized under different O_2_ concentrations. The Cu 2p spectra in Figure 3a exhibit distinct Cu 2p_3/2_ (ca. 935 eV) and Cu 2p_1/2_ (ca. 952 eV) peaks characteristic of oxidized Cu species, accompanied by shake-up satellite features, indicating the predominance of Cu^2+^ on the surface [12–13]. Meanwhile, the Rh 3d spectrum in Figure 3b shows well-defined Rh 3d_5/2_ (ca. 308 eV) and Rh 3d_3/2_ (ca. 313 eV) doublet peaks, confirming the stable incorporation of Rh^3+^ within the oxide lattice under the optimized annealing condition [14]. In contrast, under the low O_2_ concentration (5.6%), additional peaks corresponding to metallic Rh (Rh^0^) are observed at around 305 eV, indicating that the oxidative environment is insufficient to fully form the spinel CuRh_2_O_4_ phase [15]. Note that in the C 1s region (Supporting Information File 1, Figure S2), the residual carbon content in the annealed samples is negligible, suggesting that PVP is almost completely decomposed and removed under oxidative conditions at 850 °C [16]. These findings confirm that the resulting materials predominantly exist as fully oxidized metal oxides, with minimal contribution from carbon species derived from PVP.

**Figure 4:**
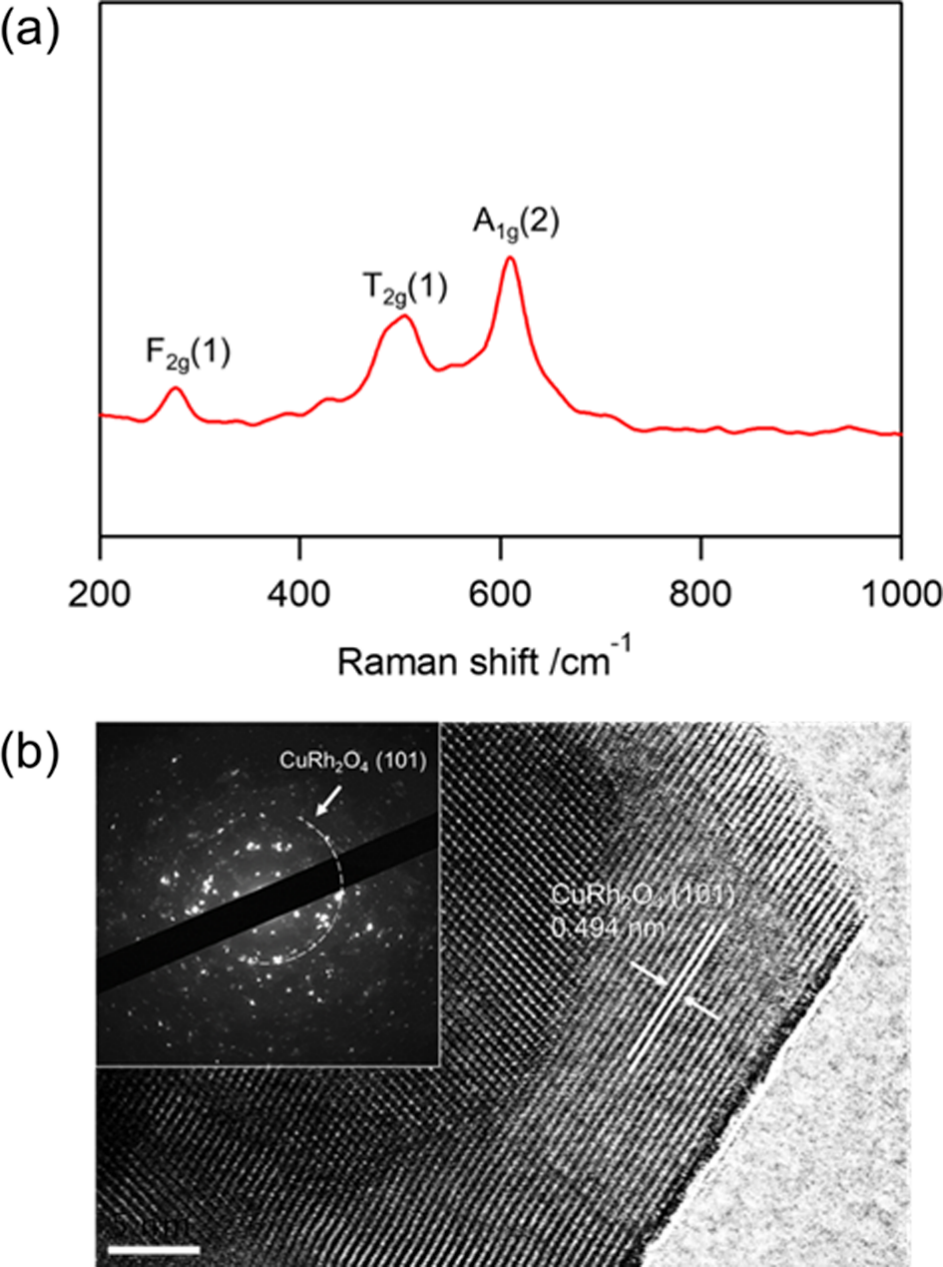
(a) Raman characterization and (b) HRTEM image of nanomaterials annealed under the O_2_ concentration of 11.1% (inset: corresponding SAED pattern).

As shown in Figure 4a, Raman spectroscopy was conducted to further examine the local bonding environments and short-range structural order of the phase-pure CuRh_2_O_4_ nanomaterials synthesized under the optimized condition (i.e., 11.1% O_2_). The spectrum exhibits characteristic vibrational modes at 277.6 cm^−1^ (F_2g_), 501.3 cm^−1^ (T_2g_) and 609.6 cm^−1^ (A_1g_), which are consistent with the spinel CuRh_2_O_4_ lattice [17–18], supporting the XRD-based phase assignment (vide supra). As shown in Figure 4b, high-resolution transmission electron microscopy (HRTEM) analysis of the electrospun CuRh_2_O_4_ nanofibers reveals clear lattice fringes with an interplanar spacing of 0.494 nm, corresponding to the (101) plane [19]. Selected area electron diffraction (SAED) patterns display ring-like diffraction features consistent with polycrystalline spinel CuRh_2_O_4_, further verifying the formation of the intended crystalline phase.

**Figure 5:**
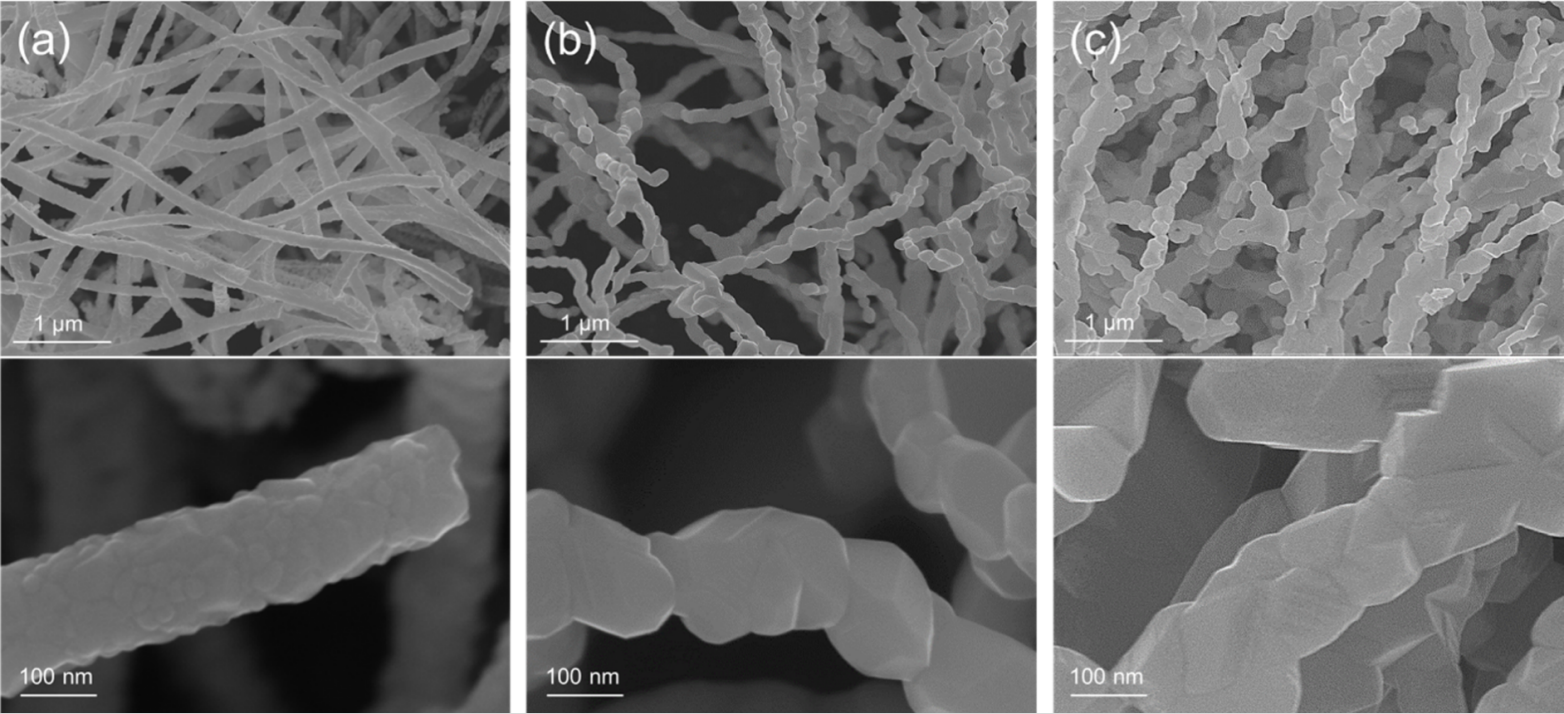
SEM images of the as-prepared nanomaterials synthesized under O_2_ concentrations of (a) 5.6%, (b) 11.1%, and (c) 22.2%.

As shown in Figure 5, scanning electron microscopy (SEM) was employed to investigate the surface morphology and structural uniformity of the electrospun nanofibers after post-annealing. In the as-spun state prior to annealing, the fibers containing PVP exhibit smooth surfaces with an average diameter of approximately 300 nm (Supporting Information File 1, Figure S3). The images show continuous and uniform nanofiber structures with well-distributed fiber networks. As the O_2_ concentration increased (from Figure 5a to Figure 5c), the surface roughness became more pronounced, suggesting that an oxygen-rich annealing atmosphere significantly affects the topology and growth behavior of the oxide nanocrystals [20]. This observation indicates that the oxidative environment influences not only phase formation but also fiber integrity and surface texture.

**Figure 6:**
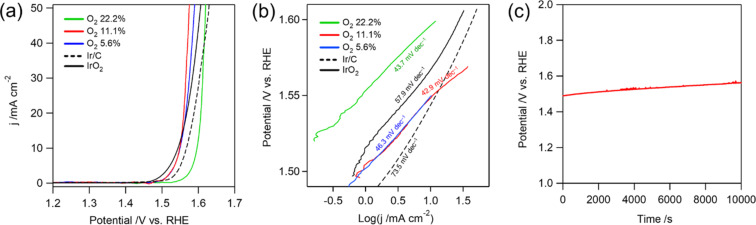
(a) LSV polarization curves of the as-prepared Cu–Rh bimetallic oxides with commercial samples (Ir/C and IrO_2_) recorded at a scan rate of 10 mV·s^−1^, (b) the corresponding Tafel plots, and (c) chronopotentiometric profile of nanofibers synthesized under 11.1% O_2_ in N_2_-saturated NaOH (aq).

The OER electrocatalytic activity of the prepared nanofibers was evaluated in N_2_-saturated 1.0 M NaOH (aq) using a standard three-electrode configuration. As shown in the *i*R-corrected linear sweep voltammetry (LSV) curves in Figure 6a, the phase-pure CuRh_2_O_4_ nanofibers prepared under 11.1% O_2_ exhibit superior OER activity compared to samples containing secondary phases, demonstrating the critical role of phase purity in catalytic performance. Notably, the optimized CuRh_2_O_4_ nanofibers outperform commercial Ir/C and IrO_2_, benchmark catalysts for alkaline OER, requiring a lower potential of 1.53 V (vs RHE) to reach 10 mA·cm^−2^ compared to 1.57 V for Ir/C and exhibiting comparable performance to IrO_2_ (1.53 V vs RHE). Furthermore, the accelerated reaction kinetics of the Cu–Rh–O series, which contribute to its enhanced intrinsic OER activity, were confirmed by Tafel analysis (η vs log *j*, where η represents the overpotential and *j* is the GSA-normalized current density) derived from the polarization curves (Figure 6b) [1]. The smaller Tafel slope observed for the sample prepared under 22.2% O_2_ (42.9 mV·dec^−1^) indicates a more favorable OER process, suggesting that the catalyst-modified electrode requires a lower overpotential to achieve higher current densities. Additionally, the electrocatalyst demonstrates excellent durability, as shown in Figure 6c, maintaining a nearly stable potential during 10,000 s of continuous OER operation at a constant current density of 10 mA·cm^−2^.

## Conclusion

In summary, Cu–Rh bimetallic single-phase spinel oxide nanofibers were successfully synthesized via electrospinning followed by post-annealing under precisely controlled oxidative environments. By deliberately regulating the O_2_ concentration in the He carrier flow, an optimized annealing condition (i.e., 11.1% O_2_) was identified to produce phase-pure CuRh_2_O_4_ nanofibers while preserving the uniform fibrous morphology. Structural and spectroscopic characterizations confirmed the formation of a highly crystalline spinel phase with well-defined nanofiber architecture; XPS analysis further verified stabilized surface chemical states of Cu and Rh under the optimized annealing atmosphere. Importantly, the phase-pure CuRh_2_O_4_ nanofibers exhibited excellent electrocatalytic activity toward the oxygen evolution reaction in 1 M NaOH. This study highlights oxygen-atmosphere engineering as a critical parameter for the reproducible synthesis and performance optimization of Cu-based spinel oxide electrocatalysts for alkaline water oxidation.

## Experimental

### Materials

Copper(II) chloride hexahydrate (CuCl_2_·6H_2_O), rhodium(III) chloride hydrate (RhCl_3_·*x*H_2_O), poly(vinylpyrrolidone) (PVP, *M*_n_ ≈ 1,300,000), sodium hydroxide (NaOH), and Nafion solution (5 wt %) were purchased from Sigma-Aldrich (St. Louis, MO, USA). Ethanol was obtained from Daejung Chemicals (Korea). Commercial Ir/C catalyst (20 wt % metal loading on Vulcan XC-72) was purchased from Premetek Co. (USA). All aqueous solutions were prepared using deionized water (resistivity ≥ 18 MΩ·cm).

### Synthesis

The electrospinning solution was prepared by dissolving Rh and Cu metal precursors at concentrations of 0.151 and 0.076 mol/L, respectively, in 2.2 mL of a mixed solvent comprising ethanol (1.5 mL) and deionized water (0.7 mL), followed by ultrasonication for 30 min to achieve complete homogenization. Subsequently, 200 mg of PVP was added to the precursor solution. The mixture was magnetically stirred for 18 h at room temperature to obtain a fully homogenized spinning solution. The prepared precursor solution was then loaded into a plastic syringe and electrospun using an electrospinning system (NanoNC, ESR200R2). Electrospinning was performed at a feed rate of 10 μL·min^−1^ with an applied voltage of 17 kV. Finally, the electrospun metal precursor/PVP nanofibers were calcined at 850 °C for 1 h with a heating rate of 10 °C·min^−1^ under a continuous mixed gas flow of O_2_ and He with controlled O_2_ concentrations (5.6%, 11.1%, and 22.2%).

### Physicochemical characterization

Morphology and elemental composition of the synthesized nanomaterials were examined using field-emission scanning electron microscopy (FE-SEM; JEOL JSM-6700F) and high-resolution transmission electron microscopy (HRTEM; JEOL JEM-2100F). Surface chemical states and crystallographic structures were analyzed by X-ray diffraction (MP-XRD; Malvern Panalytical X-ray diffractometer using Cu Kα radiation), Raman spectroscopy (HORIBA, LabRAM HR Evo 800), and angle-resolved X-ray photoelectron spectroscopy (AR-XPS; Thermo Fisher Scientific K-ALPHA XPS, Al Kα radiation at 12 kV).

### Electrochemical measurements

The as-prepared nanofibers and commercial Ir/C catalyst were separately dispersed in deionized water to obtain catalyst inks with a concentration of 2 mg·mL^−1^. An aliquot (6 μL) of each well-dispersed ink was drop-cast onto a glassy carbon (GC) disk electrode (3 mm diameter) and dried in an oven at 60 °C for 10 min. This drop-casting procedure was repeated five times, resulting in a total catalyst loading of 60 μg on each electrode. Subsequently, 10 μL of 0.05 wt % Nafion solution (diluted in ethanol) was drop-cast onto the catalyst-modified GC electrode and dried in a desiccator for 30 min. All electrochemical measurements were conducted using a standard three-electrode configuration, with the catalyst-loaded GC electrode as the working electrode, a saturated calomel electrode (SCE) as the reference electrode, and a coiled Pt wire as the counter electrode. The OER activity was evaluated by rotating disk electrode (RDE) voltammetry using an electrochemical analyzer (RDE-1 rotor/Epsilon electrochemical analyzer, BASi) in N_2_-saturated 1.0 M NaOH (aq) at a rotation rate of 1600 rpm. Current densities were calculated by normalizing the measured current to the geometric surface area (GSA) of the electrode. The GSAs were determined by chronocoulometry measurements in 0.1 M KNO_3_ containing 10 mM K_3_Fe(CN)_6_ [21]. All electrochemical measurements were performed using a CHI 920C electrochemical workstation (CH Instruments).

## Supporting Information

File 1 Additional experimental data.

## Data Availability

Data generated and analyzed during this study is available from the corresponding author upon reasonable request.
