# Supplementary material for: Oxidative atmosphere-driven formation of single-phase spinel CuRh2O4 nanofibers for alkaline water oxidation
Source: Beilstein J Nanotechnol. 2026 May 27;17:737–43. doi: 10.3762/bjnano.17.50 (PMC13224052; doi:10.3762/bjnano.17.50)
Supplement: File 1 — Additional experimental data. [file Beilstein_J_Nanotechnol-17-737-s001.pdf]

BEILSTEIN JOURNAL OF NANOTECHNOLOGY

Supporting Information

for

Oxidative atmosphere-driven formation of single-phase spinel $CuRh_2O_4$ nanofibers for alkaline water oxidation

Namhee Kim, Sumin Ko, Sohyeon Choi, Seoyoon Jang, Myung Hwa Kim and Dasol Jin

Beilstein J. Nanotechnol. **2026,** *17,* 737–743. doi:10.3762/bjnano.17.50

Additional experimental data

License and Terms: This is a supporting information file under the terms of the Creative Commons Attribution License (https://creativecommons.org/licenses/by/4.0). Please note that the reuse, redistribution and reproduction in particular requires that the author(s) and source are credited and that individual graphics may be subject to special legal provisions.
The license is subject to the *Beilstein Journal of Nanotechnology* terms and conditions: (https://www.beilstein-journals.org/bjnano/terms)

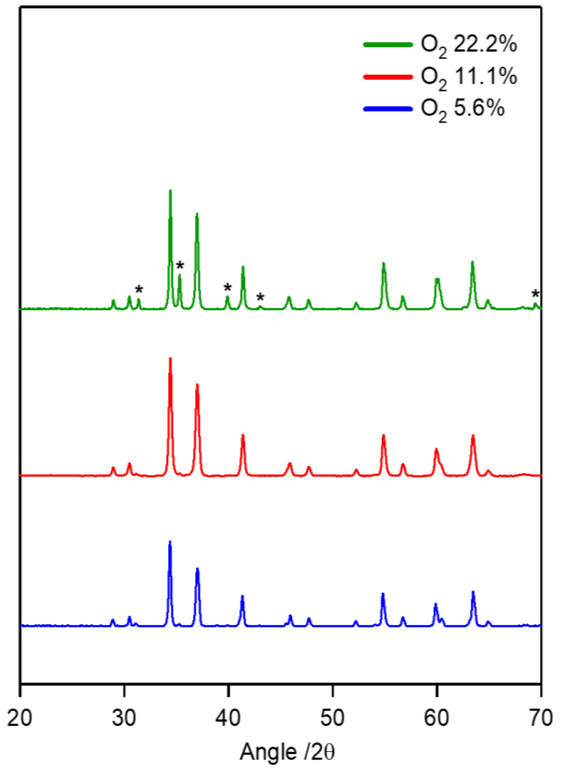

Figure S1: XRD patterns of the Cu–Rh bimetallic oxide nanomaterials synthesized under varying O_2 concentrations and annealed at 850 °C for 3 h. The unassigned diffraction peaks are attributed to the formation of spinel copper rhodium oxide ($CuRh_2O_4$), while the asterisk (*) denotes a secondary phase corresponding to $CuRhO_2$.

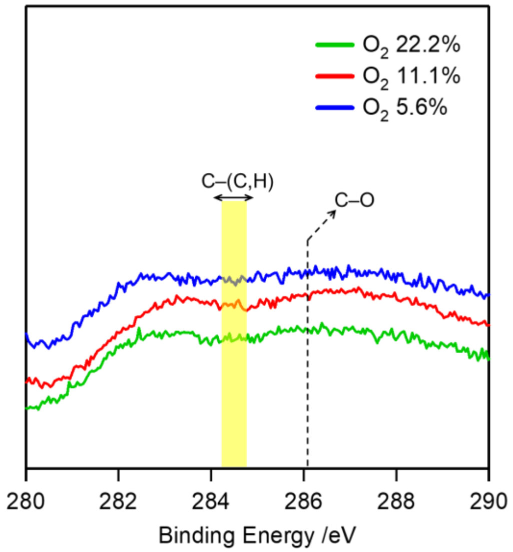

Figure S2: AR-XPS spectra of nanomaterials in the C 1s region.

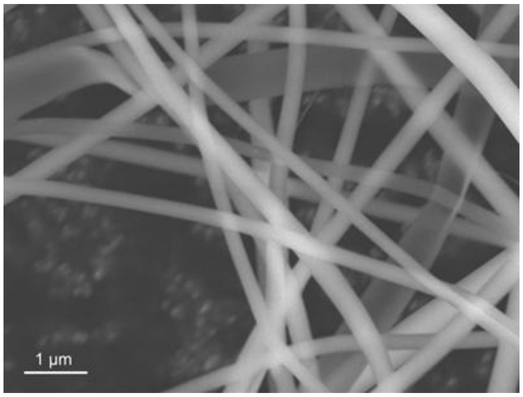

Figure S3: SEM image of the as-spun $CuCl_2$ + $RhCl_3$/PVP fibers.
